# Longitudinal piglet sampling in commercial sow farms highlights the challenge of PRRSV detection

**DOI:** 10.1186/s40813-021-00210-5

**Published:** 2021-04-12

**Authors:** Marcelo Nunes de Almeida, Cesar A. Corzo, Jeffrey J. Zimmerman, Daniel Correia Lima Linhares

**Affiliations:** 1 Department of Veterinary Diagnostic and Production Animal Medicine, College of Veterinary Medicine, Iowa State University, 1811 Veterinary Medicine Annex, 1856 Christensen Dr. Ames, Ames, Iowa 50011 USA; 2 Veterinary Population Medicine Department, College of Veterinary Medicine, University of Minnesota, St. Paul, MN USA

**Keywords:** Surveillance, Processing fluids, Family oral fluids, Serum, PRRSV, Swine

## Abstract

**Background:**

Processing fluids (PF) and family oral fluids (FOF) are population-based surveillance samples collected from 2- to 5-day-old piglets and due-to-wean piglets, respectively. Although they are described for the surveillance of PRRSV in sows and piglet populations at processing and weaning, there is limited information on their use in commercial herds. This observational study described PRRSV RNA detection over time in PF, FOF, and piglet serum collected from farrowing groups in commercial breeding farms with the objective of achieving robust, practical, and effective PRRSV surveillance protocols. Weekly PF (an aggregate sample of all litters processed in a week from each room), and FOF (a convenience sample attempted from at least 20 individual litters in at least one farrowing room each week) samples were collected from six PRRSV-endemic commercial breeding herds for up to 38 weeks. A total of 561 PF room samples, 2400 individual litter FOF samples, and 600 serum samples (120 pools of 5 samples) were collected during the study period and tested for PRRSV RNA. Data were evaluated for patterns of PRRSV RNA detection by specimen within farms over time.

**Results:**

In particular, the detection of PRRSV was commonly sporadic over time within farms (weeks of PRRSV RNA negative results followed by one or more weeks of positive results); was often non-uniform within farms (negative and positive farrowing rooms at a given point in time); and PF and FOF testing results agreement was 75 and 80% at week and room level, respectively, demonstrating that both sampling methods could complement each other. Non-uniformity in PRRSV detection in rooms sampled within the same week and detection after ≥11 consecutive weeks of PRRSV negative PF and FOF results underline the challenge of consistently detecting the virus.

**Conclusions:**

These results suggest that monitoring protocols for breeding herds attempting PRRSV control or elimination can use both PF and FOF to improve PRRSV detection in suckling pig populations.

## Background

Porcine reproductive and respiratory syndrome virus (PRRSV) has significant welfare, productivity, and economic impacts in swine breeding herds [1], for which reason producers and veterinarians implement strategies to control and/or eliminate the virus from infected herds [2]. Once a reduction in clinical signs and progress toward recovery of pre-outbreak productivity levels is achieved, it is common practice to surveil the due-to-wean piglet population as an indirect way to monitor for PRRSV in the sow herd [3].

The 2011 American Association of Swine Veterinarians breeding herd classification guidelines stated that a PRRSV stable breeding herd status is indicated by four consecutive negative samplings based on PRRSV RNA testing of serum from > 30 piglets at < 30-day intervals over 90 days [3]. If the assumptions of hypergeometric sampling hold, then sampling 30 individuals from a population should detect > 1 viremic piglet(s) when PRRSV prevalence is > 10% with 95% confidence [4]. This approach is problematic given that PRRSV prevalence and, thus, the probability of detection using 30 piglet serum samples will decline over time as effective control and/or elimination strategies achieve their intended effect. This scenario is not hypothetical, i.e., prior field research documented sustained low PRRSV prevalence (< 3%) in breeding herds attempting virus elimination [5–7].

Detection of PRRSV at low prevalence using individual animal samples, e.g., serum, is not practical in commercial production systems because the large number of samples required must necessarily incur intolerable labor and testing costs. This fact has led to the emergence of alternative population-based surveillance methods for the detection of PRRSV in piglets, e.g., processing fluids [8–11], and family oral fluids (FOF) [12, 13].

Processing fluids (PF) are defined as the serosanguineous fluid recovered from castration and tail docking tissues at the time of piglet processing [14]. The approach was first reported for antibody-based surveillance of sow farms using fluid recovered from castrated piglet tissues [15]. At the room level, Lopez et al. (2018) showed that the probability of detecting PRRSV RNA was higher for one whole-room PF compared to 30 serum samples [14]. Oral fluids (OF) have been used extensively for monitoring growing and adult pigs [16], but to a limited extent in suckling piglets. FOF is an adaptation of OF whereby a cotton rope is hung in the farrowing crate where both the dam and her piglets have access to it [12]. Almeida et al. (2020) reported a higher collection success rate for FOF versus hanging a rope where only piglets had access, likely because the piglets were mimicking the dam’s behavior (chewing) with the rope [13].

Thus, aggregate sampling approaches for infectious disease surveillance in piglet populations have been described (PF and FOF), but there is limited information on their use over time in commercial herds. This observational study described PRRSV RNA detection over time in PF, FOF, and piglet serum collected from farrowing groups in commercial breeding farms with the objective of achieving robust, practical, and effective PRRSV surveillance protocols.

## Material and methods

### Study design

This was a longitudinal study conducted in 6 PRRSV-endemic commercial breeding herds located in Iowa, Nebraska, and Minnesota, USA. Throughout the study, processing fluids (PF), family oral fluids (FOF), and serum from due-to-wean piglets were collected periodically by producers trained in sample collection. Ultimately, the decision as to the number and timing of samples was under their control. Data were evaluated in terms of patterns of PRRSV RNA detection by specimen within farms over time. This study was approved by the Iowa State University Office for Responsible Research under protocol #3-18-8730-S.

### Farms

Table 1 provides an overview of the sow herds participating in the study. Various strategies for replacement animal introduction and PRRSV immunological interventions were used by participants. Farms A and C quarantined replacement gilts (30 days) prior to introduction to the main herd, after which 10% of the group was tested for PRRSV RNA (pools of 5 serum samples) and antibody. All sows were vaccinated with a PRRSV modified live virus (MLV) vaccine over a 2-day period approximately 5 months prior to initiating the study. Farms B and D did not quarantine gilts before introduction to the main herd, but 10% of the group was tested for PRRSV RNA (pools of 5 serum samples) and antibody. All sows were vaccinated with a PRRS MLV product over a 2-day period approximately 5 months prior to initiating the study. Farm E used internal gilt replacements, i.e., no gilts were obtained from outside sources. Approximately 2.5 months prior to initiating the study, all sows were vaccinated with a PRRSV MLV product on the same day and then inoculated with live field virus 3 weeks later. Live virus inoculation (LVI) was performed using serum from naturally infected piglets (*n* = 5) on the farm. Farm F quarantined replacement gilts prior to introduction to the herd and tested 10 animals of each group of for PRRSV RNA (pools of 5 serum samples). LVI was performed on all sows in the herd 3 months prior to the initiation of the study.

**Table 1:** Characteristics of sow herds participating in the study^a^

	Farm A	Farm B	Farm C	Farm D	Farm E	Farm F
Sow inventory	3000	3000	3000	3000	6000	3300
Distance to the nearest farm	800 m	8 km	800 m	3.2 km	> 32 km	2.4 km
Farrowing rooms (crates/room)	18 (28)	15 (24)	14 (28)	20 (32)	20 (56)	13 (56)
Most recent PRRSV outbreak	18 months	6 months	6 months	6 months	3 months	5 months
PRRSV RFLP pattern^b^	1–7-4	1–10-4	1–7-4	1–7-4	1–8-4	1–7-4
PRRSV control						
- PRRSV MLV	Quarterly	Quarterly	Quarterly	Quarterly	Yes	No
- Live virus inoculation	No	No	No	No	Yes	Yes
- Shower in/shower out	Yes	Yes	Yes	Yes	Yes	Yes
- Supplies disinfected at entry	Yes	Yes	Yes	Yes	Yes	Yes
- Gilt quarantine	Yes	No	Yes	No	Yes	Yes
Surveillance specimens						
- Processing fluids (PF)						
Weeks sampled	1–19	1–19	1–19	1–19	1–38	1–18
Total PF samples	87	83	74	94	167	56
- Family oral fluids (FOF)						
Weeks sampled	9–11, 13	11–14, 16–19	13–19	12–18	3–5, 7–8, 10–12, 14–16, 18, 20–22, 25–31, 33	1–9, 11, 12, 14–17
Total FOF samples	109	480	439	573	498	301
- Serum						
Sampling period (weeks)	Not done	Not done	Not done	Not done	14, 18, 22, 24–31, 33	8, 12
Total pooled^c^ serum samples					108	12

^a^ All farms endemically infected with PRRSV, used commercial modified live vaccine (MLV) and/or live PRRSV inoculation (LVI), and practiced continuous farrowing

^b^ Based on PRRSV ORF5 sequencing

^c^ Pools of 5 serum samples

### Sample collection and processing

One PF sample per farrowing room was collected as previously described [14] from all litters being processed from piglets between three and 5 days of age. Briefly, testicles and tails, byproducts of piglet processing (castration and tail docking), were placed on a cheese cloth covering a bucket lined with a clean, disposable plastic bag. The serosanguineous fluid recovered from the tissues were transferred into a sterile 50 mL conical centrifuge tube (Fisher Scientific™, Pittsburgh, PA, USA) labeled with the date of collection and room number. PF samples were stored at the farm at − 20 °C and shipped to the veterinary diagnostic laboratory for testing on a weekly basis.

FOF were collected from due-to-wean litters in the same rooms from which PF had been collected approximately 18 days prior at approximately 21 days of age. One FOF sample was collected per litter. FOF were collected by hanging a length of 5/8-in. cotton rope (Skydog Rigging, Lake in the Hills, IL, USA) in a position that allowed access to both the sow and her piglets. Ropes were hung between 6:00 am and 7:00 am and fastened securely to farrowing crate bars using plastic zip ties. To encourage piglets to interact with the rope, the rope was unraveled and placed in such a way that the three strands hung approximately one inch from the floor. Ropes were left in place for ≥30 min, after which oral fluids were harvested by placing the wet end of the rope in a plastic bag and then pulling the rope from the bag through clenched fingers. The fluid that pooled in the bag was then poured into a 50-mL conical centrifuge tube (Fisher Scientific™, Pittsburgh, PA, USA) for transport and storage. In the laboratory, processing fluids and family oral fluids were centrifuged for 5 min at 1400 x *g* and then the supernatant submitted for PRRSV RNA testing.

Blood samples were collected using a single-use sterile BD Vacutainer™ SST™ Venous Blood Collection Tube (Thermo Fisher Scientific, Waltham, MA, USA) via jugular venipuncture. Immediately following FOF and serum collection, samples were labeled with key information: farm, collection date, farrowing crate information (position within a farrowing room). FOF and serum were held at 4–8 °C and transported to the laboratory for processing within 24 h. In the laboratory, whole blood was centrifuged for 10 min at 1600 x *g,* and then serum samples were submitted for PRRSV RNA testing.

### Sampling schedule

All samples were collected between June 2018 and January 2020 by farm staff. Sampling recommendations included: PF collection from every room being processed in a given week during the study period; FOF collection attempted from at least 20 litters from at least 1 room each week; and 30 blood samples from a convenience sampling of due-to-wean piglets from one room monthly. Table 2 provides a summary of the samples actually received from each farm by sample type.

**Table 2:** PRRSV RNA detection in processing fluid, family oral fluid, and serum samples

Farm	Processing fluids		Family oral fluids		Pooled serum (pooled by 5)	
	No. positive (Total)	No. piglets sampled	No. positive (Total)	No. piglets sampled	No. positive (Total)	No. piglets sampled
A	25 (87)	29,232	0 (109)	1199	–	
B	7 (83)	23,904	4 (480)	5280	–	
C	29 (74)	24,864	9 (439)	4829	–	
D	32 (94)	36,096	23 (573)	6303	–	
E	54 (167)	112,224	124 (498)	5478	47 (108)	540
F	0 (56)	37,632	0 (301)	3311	0 (12)	60
TOTAL	147 (561)	263,952	160 (2400)	26,400	47 (120)	600

### PRRSV RNA detection

PF, FOF, and serum samples were tested by PRRSV real-time reverse-transcriptase polymerase chain reaction (RT-rtPCR) at the Iowa State University Veterinary Diagnostic Laboratory, Ames, IA using routine procedures. Viral nucleic acids were extracted using a KingFisher® Flex automated magnetic particle processor system (Thermo Fisher Scientific) and a commercial extraction kit MagMAX™ Pathogen RNA/DNA Kit (Thermo Fisher Scientific) performed according to the manufacture’s specifications. Samples were extracted using the “high volume” chemistry. The “high volume” lysis for PRRSV extraction contained: 100 μL of sample, 120 μL of lysis/binding solution concentrate, 120 μL of 100% isopropanol, 2 μL of carrier RNA (1 μg/μL), and 2 μL of Xeno™ RNA (10,000 copies/μL) (Thermo Fisher Scientific). The high volume wash steps used 300 μL of wash buffer 1 and 450 μL of wash buffer 2.

The PRRSV RT-rtPCR was performed using the 10X PRRSV Primer Probe Mix V2 from the VetMAX™ PRRSV NA and EU kit (Thermo Fisher Scientific). The assay was modified from the original kit to use TaqMan® Fast Virus 1-Step Master Mix (4X) (Thermo Fisher Scientific) with the addition of Amplitaq 360 DNA Polymerase (Thermo Fisher Scientific). Each reaction consisted of 6.5 μL of TaqMan® Fast Virus 1-Step Master Mix (4X), 0.8 μL Amplitaq 360 DNA Polymerase (5 U/μL), 2.7 μL of nuclease-free water, 2.0 μL of the 10X PRRSV Primer Probe Mix V2, and 8.0 μL of nucleic acid template. Each plate included one positive extraction control, positive amplification control, negative extraction control, and negative amplification control. The assay was conducted on the ABI-7500 Fast system (Thermo Fisher Scientific), using the 7500 Fast System SDS Software Version 1.4.0.27. The ABI-7500 was set to run in fast mode, with cycling conditions: 5 min at 50 °C; 20 s at 95 °C; 40 cycles of 95 °C for 3 s and 60 °C for 30 s. Samples were considered positive for Ct values ≤37.

### Statistical analysis

Descriptive statistics were conducted using Microsoft Excel for Mac version 16.41 (2020). Room and week PRRSV positive status was based on ≥1 PRRSV RNA positive PF, FOF, or pooled serum samples. Crude agreement at the week level was estimated by aggregating the data from all farms at time points in which both PF and FOF samples were collected. Agreement at the week level did not take room level into account. Agreement at the room level was reported for rooms from which both PF and FOF samples were collected from the same cohort of piglets at approximately 3 and 21 days of age. Sporadic detection in a farm was defined as the change from all PRRSV RNA negative PF samples 1 week to ≥1 PRRSV RNA positive PF samples the next week.

## Results

**Fig. 1:**
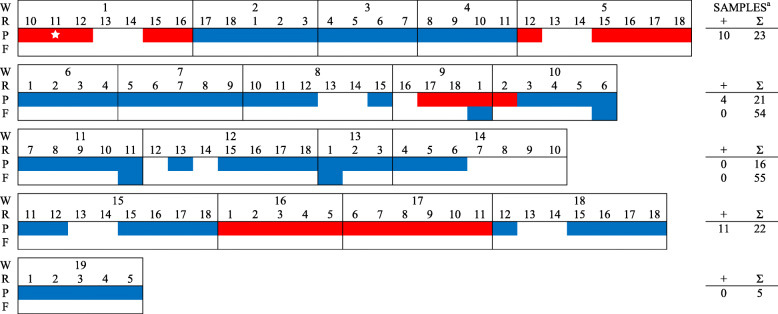
FARM A. PRRSV RNA detection (,) by week (W), farrowing room (R), and specimen (P - processing fluid, F - family oral fluid). ^a^No. RNA positive samples (+) and no. samples collected (∑).  PRRSV RFLP 1–7-4 detected

**Fig. 2:**
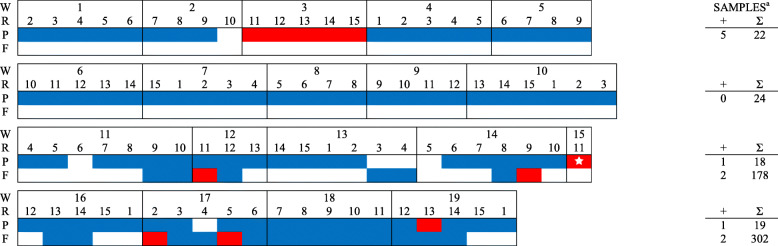
FARM B. PRRSV RNA detection (,) by week (W), farrowing room (R), and specimen (P - processing fluid, F - family oral fluid). ^a^No. RNA positive samples (+) and no. samples collected (∑).  PRRSV RFLP 1–7-4 detected

**Fig. 3:**
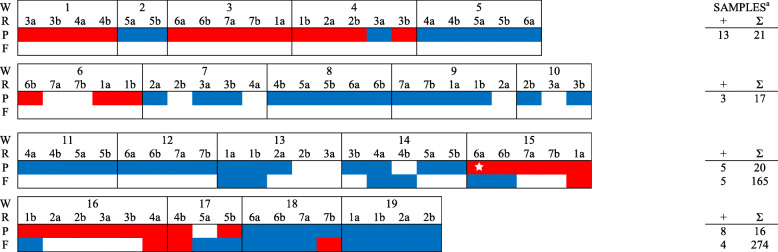
FARM C. PRRSV RNA detection (,) by week (W), farrowing room (R), and specimen (P - processing fluid, F - family oral fluid). ^a^No. RNA positive samples (+) and no. samples collected (∑).  PRRSV RFLP 1–3-2 (Fostera-like) detected. PRRSV RFLP 1–7-4 detected on week 20 (data not shown). PRRSV RFLP 1–10-4 detected on week 27 (data not shown)

**Fig. 4:**
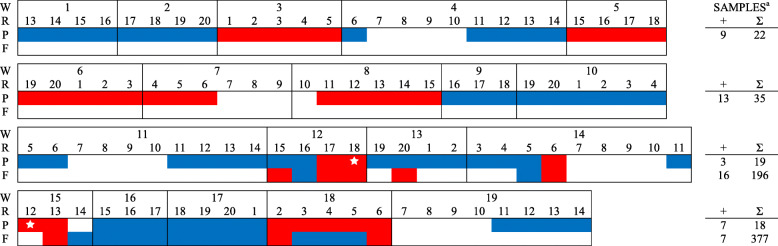
FARM D. PRRSV RNA detection (,) by week (W), farrowing room (R), and specimen (P - processing fluid, F - family oral fluid).). ^a^No. RNA positive samples (+) and no. samples collected (∑).  PRRSV RFLP 1–7-4 detected

**Fig. 5:**
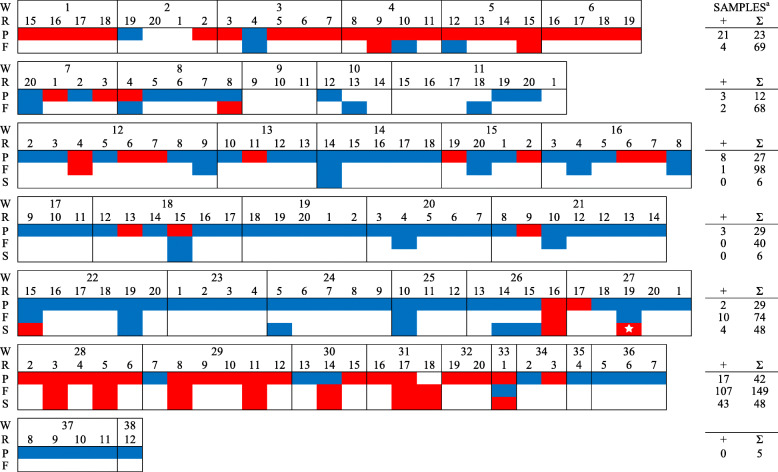
FARM E. PRRSV RNA detection (,) by week (W), farrowing room (R), and specimen (P - processing fluid, F - family oral fluid, S - Serum). ^a^No. RNA positive samples (+) and no. samples collected (∑).  PRRSV RFLP 1–8-4 detected (also at week-1, data not shown)

**Fig. 6:**
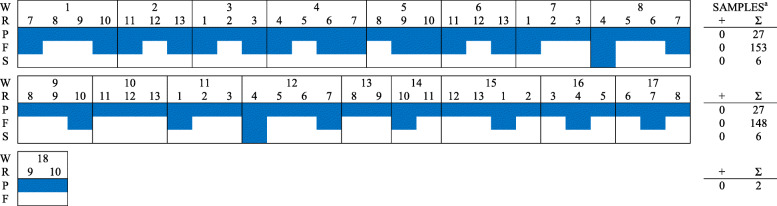
FARM F. PRRSV RNA detection (,) by week (W), farrowing room (R), and specimen (P - processing fluid, F - family oral fluid, S- Serum). ^a^No. RNA positive samples (+) and no. samples collected (∑)

In total, 561 PF samples, 2400 FOF samples, and 120 pooled piglet serum samples (5 piglets per pool) were collected from six farms during the study period and tested for PRRSV RNA (Table 2, Figs. 1, 2, 3, 4, 5, 6). Based on the productivity information from the six farms, 561 PF samples would represent ~ 264,000 piglets, and 2400 FOF would represent ~ 26,500 due-to-wean piglets. On average, PF were collected from a mean of 4.3 rooms per week and FOF from 1.9 rooms per week on each study herd. A total of 147 PF, 160 FOF, and 47 pooled serum samples from 5 farms tested positive. None of the samples (56 PF, 301 FOF, 12 pooled serum samples) from Farm F were PRRSV RNA positive. Because sampling decisions (where, when, how many) were under the control of participants, non-uniformity in sampling was observed on all farms. Incomplete data precluded some analyses or comparisons. Regardless, the results of RT-rtPCR testing revealed important information on the circulation of PRRSV at farrowing in sows and their piglets.

A comparison between PF and FOF results at the week level showed an overall agreement of 75.0% (Table 3). In PRRSV-positive farms (A - E) and including only weeks in which more than one room was sampled, PF testing revealed both negative and positive rooms in 20 of 108 PF sampled weeks, with 15 such weeks in Farm E. For FOF, 15 of 30 weeks had discordant room PRRSV status. When both PF and FOF samples were collected from the same room, discordant results between the two specimen types were observed in 23 of 114 (20.2%) times (Table 4). A comparison of serum with PF and FOF PRRSV RNA testing results showed an agreement of 76.5 and 82.4%, respectively, on a room basis (Table 5).

**Table 3:** Matched by week of collection - overall agreement in PRRSV RNA detection between processing fluids (*n* = 257 from ~ 135,936 piglets) and family oral fluids samples (*n* = 2400 from ~ 26,400 piglets) in herd A - F

		Family oral fluids	
		Positive	Negative
Processing fluids	Positive	16 (25.0%)	11 (17.2%)
	Negative	5 (7.8%)	32 (50.0%)

**Table 4:** Matched by room collected - overall agreement in PRRSV RNA detection between processing fluids (*n* = 114 from ~ 55,776 piglets) and family oral fluids samples (*n* = 2210 from ~ 24,310 piglets) in herds A - F

		Family oral fluids	
		Positive	Negative
Processing fluids	Positive	18 (15.7%)	14 (12.2%)
	Negative	9 (7.8%)	74 (64.3%)

**Table 5:** Matched by room collected - overall agreement between processing fluids (*n* = 17), serum (*n* = 102 in pools of 5), and family oral fluids (*n* = 294) samples in herds E and F

		Pooled serum	
		Positive	Negative
Processing fluids	Positive	7 (41.2%)	1 (5.9%)
	Negative	3 (17.6%)	6 (35.3%)
Family Oral Fluids	Positive	8 (47.1%)	0 (0.0%)
	Negative	3 (17.6%)	6 (35.3%)

Sporadic detection was also observed over time in both PF and FOF samples in farms A to E (Figs. 1, 2, 3, 4, 5, 6). Specific to PF, between 2 and 5 sporadic detection events were observed in each farm. The longest period of consecutive PF-negative tests was 11 weeks (Farm B, weeks 4 to 14) with a total of 51 PF (51 rooms) testing PRRSV PCR-negative. However, over this same period, 2 FOF samples were PRRSV RNA positive (weeks 12 and 14), i.e., one FOF from 22 FOF samples in one room on week 12 and one of 20 FOF in one room on week 14.

Documenting the diversity of PRRSV sequences on study farms was not within the scope of this study, but participants shared sequencing data from routine diagnostic investigations (see Figs. 1, 2, 3, 4, 5). Based on < 99% ORF5 nucleotide homology to MLV of vaccines in commercial use in the USA, Farms B, D, and E reported ≥1 wild-type PRRS viruses during the study period; Farm C reported one vaccine-like ORF5 sequence during the study and 2 wild type sequences shortly after the termination of the study (Table 6).

**Table 6:** Nucleotide homology (%) between PRRSV field isolates and commercial PRRSV modified live virus vaccines

Isolate source	RFLP^a^	Vaccine 1^b^	Vaccine 2^c^	Vaccine 3^d^	Vaccine 4^e^	Vaccine 5^f^
Farm A	1–7-4	86.9	87.9	88.7	87.3	NA^g^
Farm B	1–7-4	86.7	87.9	87.2	87.1	NA
Farm C	1–10-4	87.0	88.4	88.0	87.2	89.7
Farm D	1–7-4	87.1	87.9	87.3	87.4	NA
Farm E	1–8-4	84.3	84.3	84.4	84.1	88.9

^a^Restriction fragment length polymorphism

^b^Ingelvac PRRS ATP. Boehringer Ingelheim Vetmedica

^c^PRIME PAC PRRS® RR. Merck Animal Health

^d^Ingelvac PRRS® MLV. Boehringer Ingelheim Vetmedica

^e^FOSTERA PRRS®. Zoetis Services LLC

^f^Prevacent™ PRRS. Elanco Animal Health Inc.

^g^Not analyzed

## Discussion

Field studies have shown that PRRSV can persist in breeding herds at low prevalence, as shown in numerous examples [6, 17, 18]. Cano et al. (2008) reported that the detection of PRRSV RNA in serum from liveborn and due-to-wean piglets sampled 4 and 12 weeks after whole-herd inoculation with live PRRSV declined from 8 to 2% and 23 to 7%, respectively [17]. Kittawornrat et al. (2014) reported the detection of PRRSV RNA in 1.5% (9 of 600) of litter-based oral fluid samples collected from 4 endemically infected 12,500-sow breed-to-wean farms [6]. Vilalta et al. (2018) reported that PRRSV prevalence in piglets in a 6000-sow breeding herd ranged from 0.9 to 6.5% between 11- and 23 weeks post-outbreak [18]. Simulation studies modeled on field data predicted that PRRSV persistence in a breeding herd would depend on herd size, isolate virulence, and control practices [19–21]. As herd size increased, so did the likelihood that the virus would remain endemic, particularly in herds infected with moderately or highly virulent isolates, regardless of control strategies employed.

In this difficult disease control scenario, implementation of herd closure, MLV vaccination and/or LVI and gilt acclimatization are used to reduce sow herd prevalence, reduce the transmission of PRRSV from sows to piglets, and ultimately to eradicate the virus [2, 21, 22]. Thus, the challenge is finding a practical, cost-effective method of monitoring progress towards PRRSV elimination from breeding herds, correctly determining when within-herd transmission has ceased, and establishing when the breeding herd is truly free of the virus. Direct surveillance of breeding herds based on testing sows is not a viable option; the more practical option is to test piglet populations. Options for detecting virus at low prevalence include: 1) collecting blood samples from a large number of pigs at established intervals, e.g., 95% probability of detecting ≤2% prevalence mandates 150 samples per air space per sampling event, or 2) use ‘population-based’ sample types, such as PF or FOF, where multiple piglets are sampled at once.

PF and FOF can be used to monitor swine populations in breeding herds by sampling at processing and due-to-wean pigs, respectively. PF is an aggregate sample composed of the serosanguineous fluid recovered from tissues (testicles and tails) collected at piglet processing (2–5 days of age). The concept is similar to the tissue exudate (“meat juice”) samples previously used in *Salmonella enterica* surveillance [23], but uses only testicles collected at castration. Boettcher et al. (2010) described the use of this specimen for antibody-based surveillance of PRRSV, influenza A virus, porcine parvovirus, porcine circovirus type 2, *Mycoplasma hyopneumoniae,* and *Salmonella* [15]*.* Subsequent studies showed that PF provided a higher probability of detection than serum for the detection of PRRSV RNA at both litter and room levels [14, 24]. Collecting one PF and one serum sample per litter, Lopez et al. (2020), established true litter status by serum testing all piglets. Processing fluid detected 100% of litters when within litter prevalence was exactly 50%, while randomly sampling one piglet from the same litter resulted in 50% probability of detection. Despite higher room level sensitivity, PF results reflect PRRSV circulation in 2- to 5-day-old piglets, and negative PF results do not necessarily predict the PRRSV status of the piglet population at weaning. Thus, in the current study, 7.8% of the weeks or rooms that were PF negative were subsequently PRRSV RNA positive with FOF.

Oral fluids, a well-established sample for pathogen surveillance in the growing and adult swine population, was first described [6] for PRRSV detection in due-to-wean piglet populations by Kittawornrat et al. (2014). However, oral fluids were not widely adopted for surveillance in due-to-wean piglets due to limited success in sample collection. FOF differ from litter-based sampling in the sense that the cotton rope is hung so that both the sow and her piglets have access. The piglets, observing the dam interact with the rope, mimic her behavior, and the result is a sample representing the family unit [25]. Almeida et al. (2020) demonstrated that FOF tested for PRRSV RNA detected 100% of litters with ≥3 viremic piglets and 50% of litters with ≤2 viremic piglets [26]. Further, Almeida et al. (2020) found that 8 PRRSV RNA negative FOF samples were equivalent to collecting and testing 57 piglet serum in a population with an expected maximum PRRSV prevalence of 5%.

The problem addressed in this study is the need to correctly classify population status over the course of a PRRSV elimination program. PF and FOF are new surveillance samples that provide improved PRRSV detection surveillance in piglet populations and, by proxy, in breeding herds. However, there is as yet limited data on their use in commercial herds over time. In this study, six commercial PRRSV-endemic breeding herds undergoing PRRSV control plans including exposure to MLV or live virus inoculation used PF (~ 3 days of age), FOF, and (less frequently) serum samples to follow the spatiotemporal distribution of PRRSV in their herds.

Sampling was entirely driven by the producers, and non-uniform and/or incomplete sampling was observed on all participant farms. Incomplete sampling compromised the ability to perform the ideal statistical analyses or comparisons, but the study’s size and scope nevertheless revealed important data regarding PRRSV circulation and detection in commercial sow breeding herds. Notably, specimens (PF, FOF, or serum) collected from a farm at the same point in time and/or from the same room did not necessarily agree. Given the same specimen, different farrowing rooms tested in the same week also sometimes differed in PRRSV status. And perhaps most importantly, sporadic detection of PRRSV over time was common, e.g., PRRSV negative PF for up to 11 consecutive weeks, yet FOF collected from the same cohort at weaning were PRRSV RNA positive.

Modern sow farm facilities usually have multiple farrowing rooms, each with many farrowing crates. Discordant PF and FOF results in the same week from the same room, i.e., PRRSV RNA negative PF samples and PRRSV RNA positive OF samples or vice-versa, may be disconcerting because they provide conflicting data concerning PRRSV circulation. PF negative, followed by FOF positive, may reflect a PRRSV-negative piglet population at processing that subsequently became infected. Alternatively, PF negative may reflect low prevalence PRRSV in the piglet population, excessive pooling of tissues (> 323 piglets), a failure to include all tissues collected in the PF sample or infection of female piglets. On the other hand, PF positive, followed by FOF negative samples may result from collecting too few samples to detect the virus at a given (< 2%) prevalence or mortality in PRRSV-viremic piglets before weaning. Regardless, false-negative results can lead to the relaxation of PRRSV control and elimination practices and result in clinical outbreaks.

Non-uniformity in PRRSV detection was also observed among rooms sampled in the same week using the same specimen. This agrees with a previous study showing that the spatial distribution of viremic piglets’ is rarely homogeneous and often clustered within farrowing rooms [26]. The lack of population homogeneity poses a challenge to commonly used monitoring practices. For example, other than due-to-wean piglets (usually from a single farrowing room), the AASV PRRSV testing protocol for establishing herd status does not offer detailed guidance on selecting piglets for serum sampling [3]. By implication, the unstated assumption is that the distribution of positive piglets within different rooms is uniform and that the PRRSV status of piglets in one room will reflect the status of other rooms and the breeding herd overall.

Reports of sow farms achieving PRRSV stable status followed by detection of the historic herd PRRSV (based on ORF-5 sequencing) suggests the presence of gaps in the current herd classification protocols [7]. The current study showed that misclassification of herd PRRSV status could be avoided by implementing weekly monitoring and increasing the number of rooms sampled. However, even with weekly monitoring of more rooms, multiple weeks of negative sampling can lead to a false conclusion that the virus is no longer present in a herd. In this study, up to 11 consecutive weeks of negative PF test results were observed, followed by occasional positive PF tests thereafter. During and after the 11 PF negative weeks, occasional FOF positives were observed. These results were consistent with a previous report of sporadic PRRSV detection [7].

Overall, this study provided new information on the dynamics of PRRSV RNA detection by RT-rtPCR in PF and FOF over time (weeks) and space (rooms) and insights into the design of improved monitoring protocols for breeding herds attempting PRRSV elimination. In particular, this finding highlights the importance of continuous monitoring of piglet populations with multiple sample types to accurately detect PRRSV circulation and overcome the challenges posed by PRRSV low prevalence and persistence.

## Conclusions and implications

Processing fluids (PF) and family oral fluids (FOF) samples are highly effective population-based specimens for PRRSV RNA detection. In this study, the detection of PRRSV RNA in PF and FOF samples was often sporadic, varying week to week, and among rooms in the same sampling week. These results illustrate the challenge of confidently establishing the PRRSV status of breeding herds and underlines the fact that reliance on a single specimen (PF, FOF, or serum) or single point in time for surveillance will compromise PRRSV detection. Thus, breeding herds seeking to achieve PRRSV control and/or elimination should consider a surveillance strategy that includes collecting and testing piglet samples at processing and at weaning on a continuous basis.

## Data Availability

Restrictions apply to the availability of individual diagnostic results, and sow farm information due to producer confidentiality, and are not publicly available.
